# RNA-binding protein biomarkers NR4A2 and NR4A3 in renal ischemia–reperfusion injury diagnosis

**DOI:** 10.1097/MD.0000000000040426

**Published:** 2024-11-15

**Authors:** Junrui Chen, Ximing Chen, Keqin Zhang

**Affiliations:** a Department of Urology, The First Affiliated Hospital of Chongqing Medical University, Chongqing, China; b Urinary Nephropathy Center, The Second Affiliated Hospital of Chongqing Medical University, Chongqing, China.

**Keywords:** biomarkers, machine learning, renal ischemia and reperfusion injury, RNA-binding proteins.

## Abstract

**Background:**

The diagnosis of renal ischemia and reperfusion injury (RIRI) is crucial for renal transplant recipients. RNA-binding proteins (RBPs) may have an impact on disease development. Therefore, this study explored the biomarkers associated with RBPs in RIRI.

**Methods:**

The RIRI related datasets, GSE37838 and GSE43974, and 3964 RBPs were employed in this research. The differential expression analysis was implemented for RIRI and control to gain differentially expressed genes in GSE37838. Then, differentially expressed genes were overlapped with RBPs to acquire intersection genes. Further, the machine learning, diagnostic analysis, and expression validation were executed to filtered biomarkers for RIRI. Additionally, pathway enrichment, molecular networks, and drug prediction were proceed.

**Results:**

The area under the curve values of NR4A2 and NR4A3 were >0.7, as well as the expression trend was consistent in both datasets, and all of them were remarkably highly expressed in RIRI. Therefore, they were considered as biomarkers of RIRI. Enrichment analyses revealed that they were both associated with neuroactive ligand–receptor interactions, among others. Further, the lncRNA–miRNA–mRNA and transcription factors (TF)–mRNA network was constructed, revealing that they were all regulated by noncoding RNAs and TF, such as SNHG5-hsa-mir-10b-5p-NR4A3, CREB1, TFAP2A, etc. In addition, a large number of biomarker-related drugs were predicted, among which cadmium acetate, potassium chromate (VI), etc were associated with NR4A2 and NR4A3.

**Conclusion:**

In this study, we identified biomarkers associated with RBPs in RIRI, explored their associated pathways and drugs, which provided new insights into the clinical diagnosis and treatment of RIRI.

## 1. Introduction

Kidney transplantation is the preferred treatment for end-stage renal disease. However, renal ischemia–reperfusion injury (RIRI) is an unavoidable consequence due to the cold and warm ischemia the donor kidney experiences during procurement, storage, and transplantation.^[1]^ RIRI can lead to delayed graft function, structural damage, and systemic complications, which significantly affect both short-term and long-term outcomes for transplant recipients.^[2]^ Additionally, the shortage of kidney donors has led to an expanded donor pool that includes not only living and brain-dead donors but also kidneys from donors after circulatory death and extended criteria donors, increasing the risk of reperfusion injury in these grafts.^[3]^ Therefore, preventing or reducing ischemia–reperfusion injury is essential for enhancing the postoperative survival and quality of life of kidney transplant recipients.

RNA-binding proteins (RBPs) are crucial proteins that bind to RNA molecules via specific RNA-binding domains, playing a vital role in RNA metabolism and functional regulation. RBPs are present in both eukaryotic and prokaryotic organisms and are essential in various gene expression processes, including posttranscriptional regulation, RNA splicing, transport, stability, and translation.^[4]^ Research indicates that defects in RBP function and changes in their expression are linked to various diseases, such as neurological disorders, muscle atrophy, and cancer. RBPs are also associated with several kidney diseases.^[5]^ For instance, human antigen R (HuR) has been shown to have renal protective functions in acute kidney injury (AKI), though it may also contribute to glomerulosclerosis, tubulointerstitial fibrosis, and diabetic kidney disease.^[6]^ The loss of Bicaudal C (Bicc1) is linked to cystic kidney diseases,^[7]^ and Y-box binding protein 1 (YB-1) is involved in the pathogenesis of AKI, diabetic kidney disease, and glomerular diseases.^[8]^ Growing evidence suggests that targeting RBPs and their interactions with RNA may offer promising strategies for treating kidney diseases. However, research on RBPs related to RIRI remains limited, and their mechanisms of action in this condition are not well understood.

Few studies have explored the role of RBPs in RIRI. Therefore, in this study, we used data from public databases related to RIRI and applied comprehensive bioinformatics approaches to identify RBP-related biomarkers in RIRI. Using machine learning and expression profiling, we constructed a molecular interaction network and predicted potential drug targets for these biomarkers. This research offers new perspectives and insights into RBP-focused studies in the context of RIRI, which is crucial for further understanding the pathogenesis of this condition.

## 2. Materials and methods

### 2.1. Data source

The RIRI related datasets were mined from Gene Expression Omnibus database (https://www.ncbi.nlm.nih.gov/geo/). The sequencing data of GSE37838 and GSE43974 were gained via GPL570[HG-U133_Plus_2] Affymetrix Human Genome U133 Plus 2.0 Array and GPL10558 Illumina HumanHT-12 V4.0 expression beadchip, respectively, which contained 78 (70 RIRI and 8 control) and 391 (203 RIRI and 188 control) renal tissue samples separately. Then, 3964 RBPs were collected from previous study (Table S1, Supplemental Digital Content, http://links.lww.com/MD/N865).^[4]^

### 2.2. Differential expression and enrichment analysis

The differential expression analysis was implemented via limma (v3.54.1) for RIRI and control to gain differentially expressed genes (DEGs). The |log_2_FC| > 0.5 and *P* value < .05 were filter conditions. The volcano map and heatmap of DEGs were presented via ggplot2 (v3.4.1) and ComplexHeatmap (v2.14.0), respectively. Then, the DEGs were overlapped with RBPs to acquire intersection genes. Further, enrichment analysis was executed using clusterProfiler (v4.2.2) to probe pathway and function related to these genes, including Gene Ontology and Kyoto Encyclopedia of Genes and Genomes (*P* < .05).

### 2.3. Machine learning and expression verification

On the basis of the intersection genes, setting family = binomial, LASSO analysis was conducted using glmnet (v4.1-4), and the best lambda value was selected at the smallest error to obtain the feature genes. Then, Boruta analysis was utilized to filter the feature genes for the intersection genes using Boruta (v8.0.0). Subsequently, the feature genes common to both methods were selected as candidate genes. The receiver operating characteristic curves of the candidate genes were plotted by pROC (v1.18.0), and the genes with area under the curve (AUC) values >0.7 were taken as candidate biomarkers. The expression of these genes was extracted in GSE37838 and GSE43974, and their expression differences between disease and control were compared by Wilcoxon (*P* < .05). Those with consistent expression trends in both datasets and marked differences between groups were finally selected as biomarkers.

### 2.4. Pathway enrichment and GeneMANIA

To clarify the pathways in which biomarkers were involved in RIRI, gene set enrichment analysis (GSEA) was executed using clusterProfiler (v4.2.2). Firstly, correlation coefficients between each biomarker and other genes were calculated and sorted in a decreasing order. The c2.cp.v7.2.symbols.gmt was retrieved from The Molecular Signatures Database (MSigDB, https://www.gsea-msigdb.org/gsea/msigdb) as the background gene set, and then GSEA was conducted (*P* < .05). The top 5 most notable pathways were selected for visualization. Based on the GeneMANIA database (http://genemania.org/), gene–gene interaction network was established for probing biomarker functionally similar genes and clarifying their related functions.

### 2.5. Molecular networks and drug prediction

To investigate the regulatory role of biomarkers involved in RIRI, we constructed lncRNA–miRNA–mRNA and transcription factors (TF)–mRNA networks. Firstly, miRTarBase v8.0 database (https://mirtarbase.cuhk.edu.cn) was employed to retrieve biomarker-targeted miRNAs. Then, the miRNA–corresponding lncRNAs were predicted in miRNet database (https://www.mirnet.ca/miRNet/home.xhtml), and subsequently, Cytoscape (v3.9.1) was utilized to construct the lncRNA–miRNA–mRNA regulatory network. In addition, the TF of the biomarkers were predicted using JASPER database (https://www.networkanalyst.ca/), and the TF–mRNA network was mapped. In addition, the protein–chemical interactions (ProtChemSI) database (http://pcidb.russelllab.org) was exploited to predict biomarker-associated drugs, and biomarker–drug network was established via Cytoscape.

## 3. Results

### 3.1. Identification and function enrichment of 16 intersection genes

**Figure 1.**
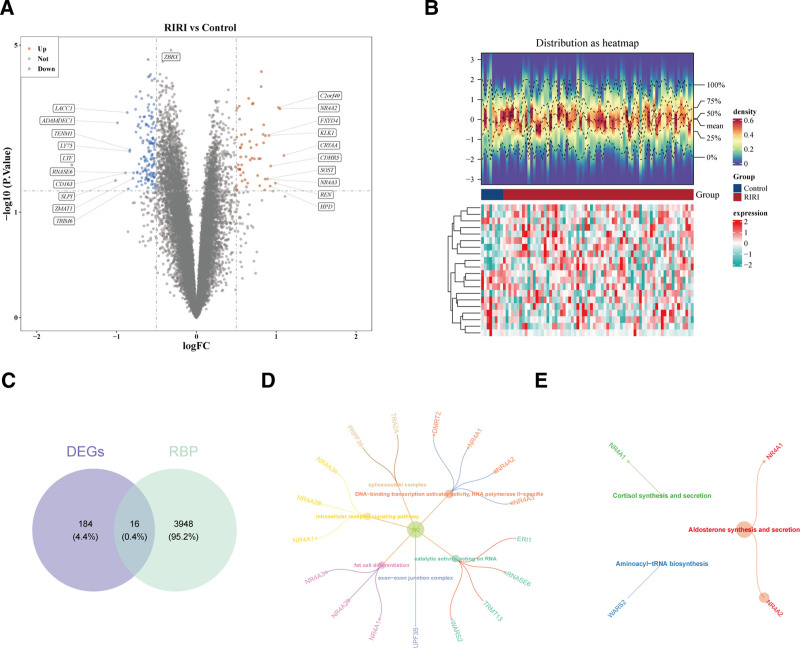
(A) Volcano plot for DEGs. (B) Heatmap for DEGs. (C) Venn diagram for RBP-RGs. (D and E) GO/KEGG enrichment of RBP-RGs. DEGs = differentially expressed genes, RBP-RGs = RNA-binding proteins related genes, GO = Gene Ontology, KEGG = Kyoto Encyclopedia of Genes and Genomes.

The 200 DEGs were gained between RIRI and control, which contained 55 up-regulated and 145 down-regulated genes (Fig. 1A and B). The 16 intersection genes were filtered via overlap of DEGs and RBPs (Fig. 1C). Further, 243 Gene Ontology items, including 201 biological process, 8 cellular component and 34 molecular function, such as DNA-binding transcription activator activity, RNA polymerase II-specific, exon–exon junction complex, fat cell differentiation, catalytic activity, acting on RNA, etc, and 3 Kyoto Encyclopedia of Genes and Genomes pathways were enriched, including cortisol synthesis and secretion, aldosterone synthesis and secretion, aminoacyl-tRNA biosynthesis (Fig. 1D and E).

### 3.2. NR4A2 and NR4A3 considered as biomarkers for RIRI

**Figure 2.**
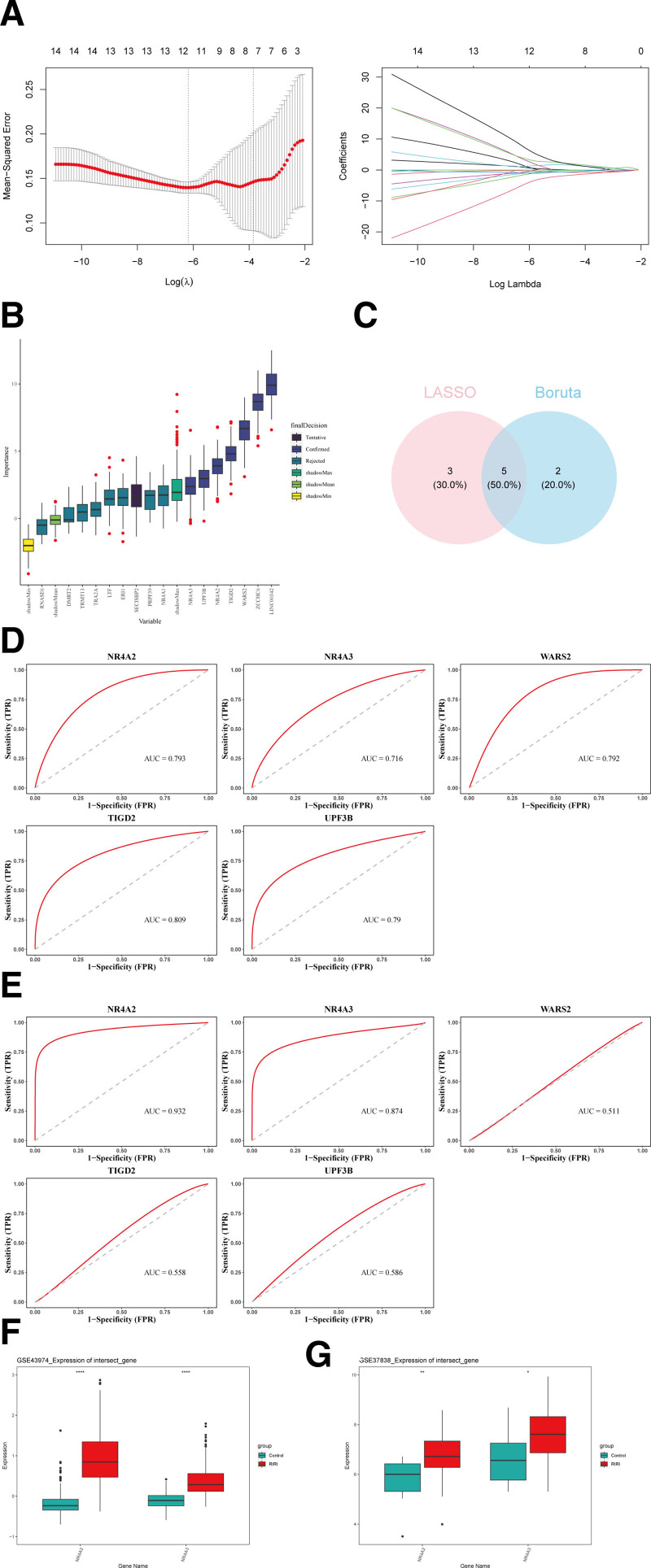
(A) λ selection diagram in Lasso model and LASSO logistic coefficient penalty plot. (B) Boruta algorithm importance ranking. (C) Venn diagram for 2 machine-learning ways. (D) ROC curve of characteristic genes in the training set. (E) ROC curve of characteristic genes in the validation set. (F and G) The difference in expression of candidate biomarkers between the disease group and the control group in the training/validation set. ROC = receiver operating characteristic.

A total of 8 and 7 signature genes were filtered by LASSO (Fig. 2A) and Boruta (Fig. 2B) analyses, respectively, and 5 of them were common genes, namely NR4A2, NR4A3, WARS2, TIGD2, and UPF3B, as candidate genes (Fig. 2C). In GSE37838 and GSE43974, the AUC values of NR4A2 and NR4A3 were >0.7, indicating high diagnostic performance for RIRI, which can be considered as candidate biomarkers (Fig. 2D and E). Moreover, they showed consistent expression trends in both datasets and were remarkably highly expressed in the RIRI group (*P* < .05) (Fig. 2F and G).Therefore, NR4A2 and NR4A3 were adopted as biomarkers for RIRI.

### 3.3. Biomarkers participated in complex pathways and functions

**Figure 3.**
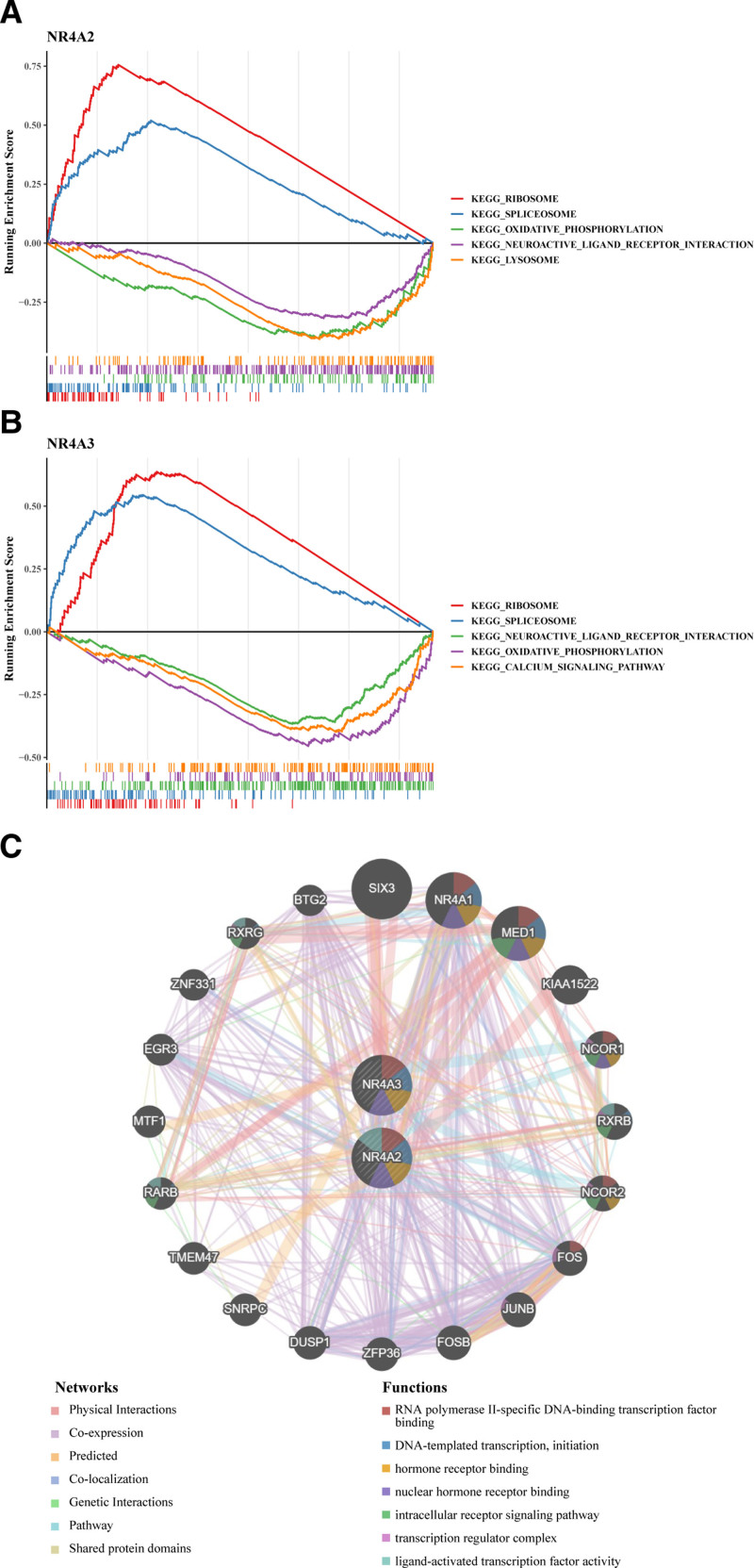
(A) GSEA enriched pathways of NR4A2. (B) GSEA enriched pathways of NR4A3. (C) Analysis of biomarkers and their co-expressed genes by GeneMANIA. GSEA = Gene Set Enrichment Analysis.

We further explored the biomarker-associated pathways and functions that may influence the development of RIRI. GSEA results showed that both biomarkers were prominently associated with ribosome, spliceosome, oxidative phosphorylation, and neuroactive ligand–receptor interaction (Fig. 3A and B). In addition, the GeneMANIA database predicted 20 genes with similar functions to the biomarkers, and then, a GGI network containing 22 genes as well as 167 links was constructed, and we found that both NR4A2 and NR4A3 were associated with RNA polymerase II-specific DNA-binding transcription factor binding, DNA-template transcription, initiation, intracellular receptor signaling pathway, nuclear hormone receptor binding, etc (Fig. 3C).

### 3.4. Discussed molecular mechanisms and potential drugs of biomarkers

**Figure 4.**
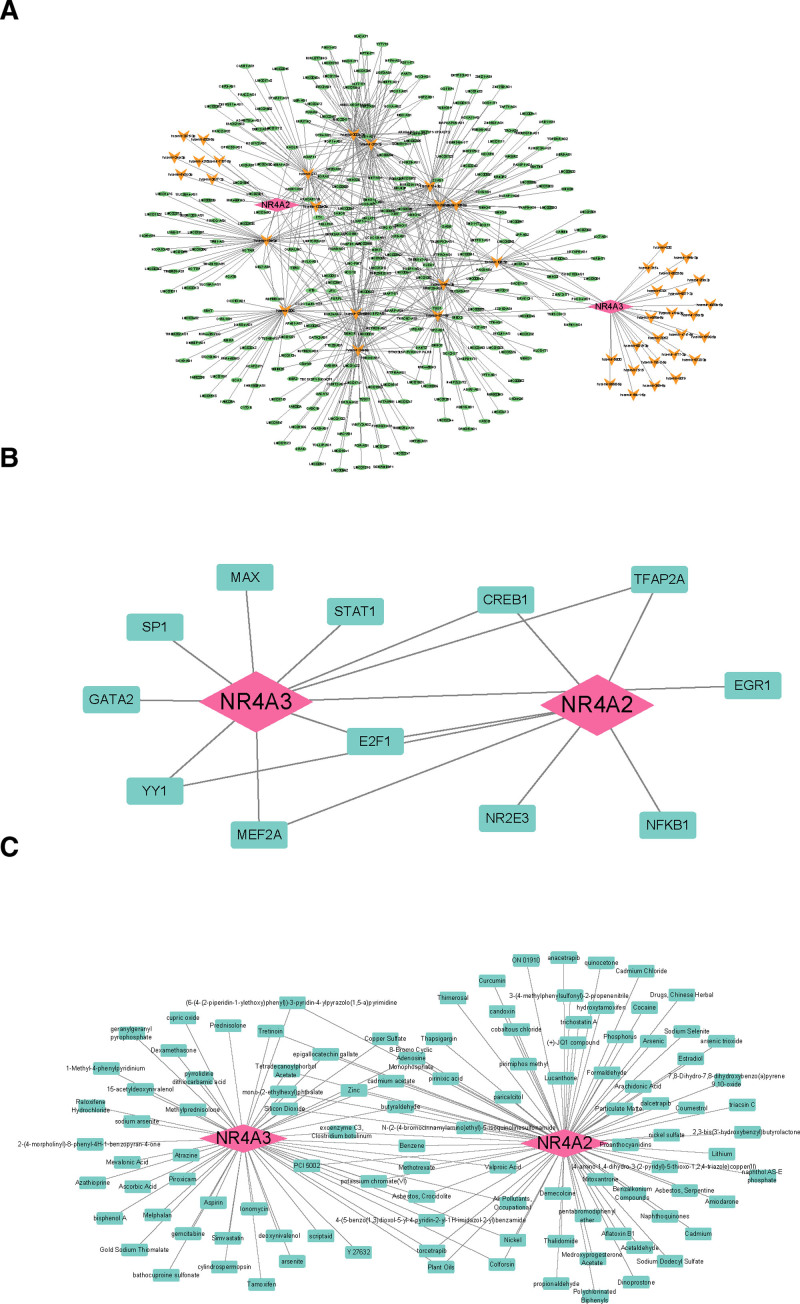
(A) lncRNA–miRNA–mRNA regulatory network of NR4A2/NR4A3. (B) Transcription factor network of NR4A2/NR4A3. (C) Drug prediction network of NR4A2/NR4A3.

Corresponding miRNAs and lncRNAs were predicted from the miRTarBase v8.0 database and the miRNet database, respectively. The lncRNA–miRNA–mRNA network containing 381 points and 658 edges was structured using Cytoscape, e.g. SNHG5-hsa-mir-10b-5p-NR4A3, GPRC5D-AS1-hsa-mir-217-NR4A2, and so on (Fig. 4A). In addition, the corresponding TF of the biomarkers were predicted from JASPER database, and a TF–mRNA network with 14 points and 17 edges was constructed, in which CREB1, TFAP2A, E2F1, YY1, and MEF2A were the common TF (Fig. 4B). They were also demonstrated that the biomarkers were all regulated by both the noncoding RNAs and the TF to affect the RIRIs. Furthermore, in this study, the corresponding drugs of a large number of biomarkers were predicted. The gene–drug network with 110 points and 132 edges was established, in which there were multiple drugs associated with both NR4A3 and NR4A2, such as (6-(4-(2-piperidin-1-ylethoxy)phenyl))-3-pyridin-4-ylpyrazolo(1,5-a) pyrimidine, cadmium acetate, potassium chromate (VI), etc (Fig. 4C).

## 4. Discussion

RIRI is a leading cause of AKI,^[9]^ commonly occurring during major surgeries, shock, or organ transplants.^[10]^ Recently, the involvement of RBPs in this injury has gained significant attention. Various studies have demonstrated that RBPs, including CIRP and HuR, play crucial roles in RIRI by modulating inflammation and apoptosis. Tom et al demonstrated that cold-inducible RNA-binding protein (CIRP) worsens RIRI. CIRP-deficient mice (Cirp-/-) had lower blood urea nitrogen and creatinine levels post-reperfusion, along with significantly reduced inflammatory markers like IL-6 and cyclooxygenase-2. This indicates that CIRP significantly exacerbates kidney damage and inflammation.^[11]^ Conversely, Ayupova et al found that the RNA-binding protein HuR provides protection against ischemia–reperfusion injury. Suppressing HuR expression increased apoptosis in mouse renal tubular cells under ischemic stress, while overexpressing HuR reduced apoptosis, promoting cell survival and mitigating kidney injury.^[12]^ These findings suggest that therapies targeting these RBPs could offer novel strategies for reducing RIRI.

Our study identified NR4A2 and NR4A3 as biomarkers for RIRI using differential analysis, machine learning, and diagnostic evaluations. We found these biomarkers involved in pathways related to ribosome function and neuroactive ligand–receptor interactions. Additionally, we developed molecular regulatory networks linked to these biomarkers and explored related drugs like valproic acid, which may hold potential for diagnosing RIRI.

We identified NR4A2 and NR4A3 as RBP-related biomarkers with diagnostic significance for RIRI. Both NR4A2 (Nurr1) and NR4A3 (Nor1) belong to the nuclear receptor subfamily 4A (NR4A), which includes NR4A1 (Nur77).^[13]^ These receptors act as transcription factors and are integral to processes such as cell cycle regulation, apoptosis, inflammation, and metabolism.^[14]^ They are also vital in tumor suppression, immune responses, and cardiovascular function, with implications for cancer therapy.^[15]^ NR4A2 and NR4A3 are early response genes activated under stress, contributing to cellular homeostasis and pathophysiological processes.^[16]^ However, the role of NR4A family proteins in RIRI has not yet been explored in the literature.

In our study, NR4A2 and NR4A3 showed AUC values of 0.793 and 0.716 for RIRI, respectively, with values of 0.932 and 0.874 in the validation cohort, indicating superior diagnostic capabilities compared to earlier studies. Both biomarkers were significantly overexpressed in the disease group, consistent with dataset analyses and animal experiments. Although research on NR4A2 and NR4A3 in RIRI is limited, their roles in other ischemic injuries suggest that their protective mechanisms might also be relevant to RIRI. In cardiovascular and neural ischemic injuries, these biomarkers influence IRI by modulating oxidative stress responses and apoptotic pathways.^[17]^ Therefore, while direct studies on NR4A2 and NR4A3 in RIRI are lacking, their functions in related conditions underscore their potential as therapeutic targets for RIRI, warranting further mechanistic research.

GSEA analysis linked these biomarkers to pathways including ribosome function, neuroactive ligand–receptor interaction, spliceosome activity, and oxidative phosphorylation. These processes likely involve complex molecular and cellular mechanisms in RIRI. For instance, ischemia-induced impairment of oxidative phosphorylation reduces ATP, disrupting ribosome function and protein synthesis. During reperfusion, ROS generation may activate neuroactive ligand receptors, triggering inflammation and apoptosis. Additionally, alterations in RNA splicing could impact cellular responses and repair capacity. Understanding these connections is vital for developing effective therapeutic strategies to mitigate RIRI.

We also predicted several drugs related to these biomarkers, including valproic acid, cadmium acetate, benzene, and methotrexate. Valproic acid, an antiepileptic drug used to treat epilepsy, bipolar disorder, and other neurological conditions, increases gamma-aminobutyric acid (GABA) levels in the brain.^[18]^ Some studies suggest it has protective effects on the kidneys, particularly by reducing oxidative stress and inhibiting inflammation.^[19]^ Although the specific mechanisms of valproic acid in RIRI remain under investigation, its potential protective role is significant.^[20]^ Our study provides valuable insights into the possible role of valproic acid in kidney diseases, aiding further exploration of its specific mechanisms in RIRI.

Despite its contributions, this study has several limitations. First, the limited database resources and sample size may introduce biases in the diagnostic model’s accuracy. Additionally, our drug prediction results have not been experimentally validated, necessitating cautious interpretation and validation through in vivo and in vitro experiments in future research. Furthermore, since our experiments are based on animal models, they can only suggest the potential diagnostic value of NR4A2 and NR4A3. As no clinical trials were conducted, the definitive diagnostic accuracy of NR4A2 for RIRI cannot be asserted. Therefore, the conclusions of this study should be interpreted with caution.

## 5. Conclusion

In this study, we identified NR4A2 and NR4A3 as key biomarkers associated with RNA-binding proteins in RIRI. Their significant differential expression suggests potential for clinical diagnosis. We also explored relevant pathways and constructed regulatory networks involving noncoding RNAs and transcription factors. Additionally, potential drug candidates linked to these biomarkers were identified. These findings enhance our understanding of RIRI and may improve diagnostic and treatment approaches for renal transplant recipients.

## Acknowledgments

We are grateful to all public database, and all participants in our study.

## Author contributions

**Conceptualization:** Junrui Chen.

**Data curation:** Junrui Chen.

**Formal analysis:** Junrui Chen.

**Funding acquisition:** Keqin Zhang.

**Investigation:** Ximing Chen.

**Methodology:** Ximing Chen.

**Resources:** Ximing Chen.

**Software:** Junrui Chen.

**Supervision:** Keqin Zhang.

**Validation:** Junrui Chen.

**Visualization:** Junrui Chen.

**Writing – original draft:** Junrui Chen.

**Writing – review & editing:** Ximing Chen.

## Supplementary Material
